# Detection of Pathological Changes in the Aorta during Thoracic Aortic Aneurysm Progression on Molecular Level

**DOI:** 10.1155/2017/9185934

**Published:** 2017-10-12

**Authors:** Miroslava Rabajdová, Peter Urban, Ivana Špaková, Artemiou Panagiotis, Michaela Ferenčáková, Dušan Rybár, Nikita Bobrov, František Sabol, Mária Mareková

**Affiliations:** ^1^Department of Medical and Clinical Biochemistry, Faculty of Medicine, Pavol Jozef Šafárik University, Košice, Slovakia; ^2^Department of Heart Surgery, Faculty of Medicine, Pavol Jozef Šafárik University and VUSCH, Košice, Slovakia; ^3^Department of Anaesthesiology and Resuscitation, Faculty of Medicine and VUSCH, Košice, Slovakia; ^4^Department of Forensic Medicine, Pavol Jozef Šafárik University, Košice, Slovakia

## Abstract

The progression of thoracic aortic aneurysm depends on regulation of aortic wall homeostasis and on changes in the structural components of the extracellular matrix, which are affected by multiple molecular signalling pathways. We decided to correlate the diameter of ascending thoracic aneurysm with gene expression of inflammation markers (IL-6, CRP), cytokine receptors (IL-6R, TNFR1, and TNFR2), and extracellular matrix components (Emilin-1, MMP9, and TIMP) for detection of the degree of pathological process of TAA formation. The experimental group was divided into three groups according to the diameter of the aortic aneurysm. Whole blood and tissue samples were properly collected and used for nucleic acid, chromatin, and protein isolation. The mRNA levels were detected by qRT-PCR. For the detection of protein levels a Cytokine Array IV assay kit was used in combination with a biochip analyzer. In aortic tissue, significant positive correlations were found between increased mRNA levels of inflammatory cytokines (CRP and IL-6) on both mRNA levels in tissue and protein from the blood with maximum in stage 3. Changes of gene expression of selected genes can be used for the experimental study of the inflammatory receptor inhibitors during trials targeted on slowing down the progress of aortic wall aneurysm.

## 1. Introduction

Thoracic aorta diseases are among the most common causes of death in the US and other developed countries. The incidence of thoracic aortic aneurysm has been estimated at 6 cases per 100,000 persons per year [1], and its prevalence has been estimated at 3-4% in patients over age 65 years old. According to possible endovascular therapy (EVT) or its combination with surgical therapy, it seems most practical to divide thoracic aorta diseases into acute aortic syndromes (AAS) and chronic aneurysmal transformation (thoracic aortic aneurysm—TAA) [2]. The AAS group includes penetrating aortic ulcer (PAU), bordered aortic intramural hematoma (IMH), and classical acute aortic dissection, while chronic aneurysmal transformation includes right degenerative aneurysm of the ascending or descending aorta, false TAA (pseudoaneurysm), and posttraumatic TAA [3].

The growth rate of TAA varies by lesion location, with ascending aneurysms growing at a rate of 0.07 cm per year. The rate of growth has also been demonstrated to increase with aneurysm size [4]. When the diameter of the thoracic aorta is more than 3.5 cm, or twice the normal diameter, the thoracic aorta is considered aneurysmal. The risk of aneurysm rupture is proportionate to its size [5]. The decision of whether to surgically treat a patient is based on aneurysm size and patient operative risk. Annual risk of rupture, dissection, or death is 14.1% in patients with aneurysms larger than 6 cm, compared with 6.5% for aneurysms between 5 and 6 cm [6]. Additionally, repair is suggested in patients with documented aneurysm growth of >1 cm per year [7]. The progression of aortic aneurysm probably results from a combination of chronic inflammation, hemodynamic stress, aortic mechanical injury, and epidemiologic risk factors. Aortic functions as well as regulation of aortic wall homeostasis depend on changes in the structural components of the extracellular matrix (ECM), which are affected by multiple molecular signalling pathways.

Microfibrils, as the main structural component of aortic wall ECM, provide a scaffold for the lysyl oxidase protein family to cross-link tropoelastin monomers to form mature elastic fibres [8]. Microfibrils are composed mainly from fibrillin and several microfibril-associated proteins (elastin microfibril interface-located protein 1 (Emilin-1), microfibril-associated glycoproteins (MAGP-1,2), and fibulins) [9]. Emilin-1 is known to be a binding precursor of TGF-*β*, called pro-TGF-*β*, and inhibits its maturation by furin convertases [10]. Defects in Emilin-1 expression affect the formation and function of elastic lamellae, increasing the degree of inflammation.

Inflammation participates in the pathogenesis of aortic aneurysm formation by the migration of T-lymphocytes and macrophages diffusely throughout the adventitia to the tunica media [11]. Several cytokines and chemokines that are produced during their transfer (TNF-*α*, interferon-*γ*, IL-1, IL-2, IL-6, and IL-8) are upregulated and promote the recruitment of other inflammatory cells to the aortic wall [12]. The main protein of the acute phase of inflammation—serum C-reactive protein (CRP)—has been reported in patients with stenotic atherosclerotic disease and is associated with an increased risk of developing cardiovascular events [13]. However, only a few articles have been published describing serum CRP and IL-6 in patients with TAA. Inflammatory changes consequently induce the secretion of matrix metalloproteinases (MMP), elastase, and collagenase from macrophages and neutrophils that are capable of directly degrading the ECM and may also contribute to the detachment of smooth muscle cells from the ECM, leading to cell death [14]. The important regulators of local MMP activity are tissue inhibitors of metalloproteinases (TIMPs) [15]. Animal data support a role for MMP and TIMP in the development of aortic aneurysm, especially in Marfan syndrome [16]. Most of the data on MMPs and aortic aneurysm are from studies of abdominal aortic aneurysms, while data is lacking on MMP in TAA. Studies of TAA have focused on changes in genetic expression, which does not necessarily translate into the changes in protein expression that determine the effect on the aorta [17]. The proteolytic theory of TAA development envisions increased concentrations of MMP and reduced concentrations of TIMP in the aorta acting in concert to increase ECM degradation, leading to an aorta that is more likely to expand in response to the hemodynamic load [18]. In this study, we decided to correlate the diameter of ascending thoracic aneurysm with gene expression of inflammation markers (IL-6, CRP), cytokine receptors (IL-6R, TNFR1, and TNFR2), and components of ECM (Emilin-1, MMP9, and TIMP) for detection of the degree of pathological process of TAA formation. The obtained results could help surgeons decide if the progression of aortic aneurysm is too fast and give them a chance to improve the lifetime and healthcare of patients suffering with progressive TAA.

## 2. Materials and Methods

### 2.1. Experimental Groups and Sample Collection

The experimental group (*n* = 60) consisted of patients suffering from thoracic aortic aneurysm, regurgitation, and aortic valve stenosis, who were divided into three groups according to the diameter of the aortic aneurysm (stage 1: 43 ± 2.3 mm, stage 2: 51 ± 2.8 mm, and stage 3: 59.5 ± 3.7 mm). Each group was characterized by age, gender, BMI, presence of aortic regurgitation or aortic stenosis (AR/AS), bicuspid or tricuspid aortic valve (BAV/TAV), hypertension, diabetes mellitus, current smoking, and family predispositions for cardiovascular diseases (Table 1). Results of preoperative medical tests of individual biochemical and haematology biomarkers were within acceptable physiology intervals according to the patient's clinical status.

Whole blood was collected from all patients of the experimental group during the standard preoperative examination done in cooperation with the Eastern Slovak Institute of Cardiovascular Diseases (VUSCH). Results of preoperative medical tests of individual biochemical and hematology biomarkers were within acceptable physiology intervals according to the patient's clinical status. The tissue samples of patients in the experimental group were collected during the following surgical procedures: replacement of the ascending aorta, valve-sparing procedure (aortic root remodeling), and replacement of the aortic valve (AVR) with a mechanical or biological prosthetic valve. Other concomitant procedures were replacement of the mitral valve (MVR) and off-pump coronary artery revascularization (OPCAB). The operations were performed through a median sternotomy with the use of cardiopulmonary bypass, mild hypothermia, and cardioplegic arrest. Cardio anesthesia was performed according to the standard protocol.

### 2.2. Control Group and Sample Collection

Control group (*n* = 35) blood samples were taken from blood donors in cooperation with the UNLP Department of Haematology and Transfusiology. The control group was composed of people with negative results from biochemical and haematological screening medical tests. Donors declared themselves free of any symptoms of cardiovascular diseases, and their clinical imaging methods, ultrasonography and computed tomography angiography, were also negative. Control materials of the ascending part of thoracic aortic tissues (*n* = 10) were obtained from the Department of Forensic Medicine of LF UPJŠ and UNLP. These necroptic samples were collected immediately after death. Cause of deaths was not related to pathology, such as thoracic aortic aneurysm, regurgitation or stenosis of the aortic valve, or any pathology associated with myocardial damage. Histopathology detection of the myocardial tissue samples did not show any noticeable changes.

All clinical investigations using human samples have been carried out in accordance with the code of ethics of the world medical association (Declaration of Helsinki). Healthy subjects in the control group and patients in the experimental group answered a medical sheet and questionnaire. Patients were informed by their doctor about the use of their blood and tissue for experimental diagnostic purposes. Informed consents were signed. Ethical consent for this study was given by the institutional committee on human research, was approved by the ethical committee of the Eastern Slovak Institute of Cardiovascular diseases (VUSCH), and was compliant with ethical standards on human experimentation and with the Declaration of Helsinki.

### 2.3. RNA, DNA, and Chromatin Isolation

All tissue samples were frozen immediately after harvesting at −196°C and stored in a freezer at −80°C. Whole blood samples, collected into Paxgene test tubes, were stored in a fridge for 2 hours and then used for isolation of DNA and RNA. For isolation of total RNA from 40 mg of tissue or 2 ml of whole blood, the isolation protocol RNeasy Mini Kit (Qiagene, Hilden, Germany) was used following the manufacturer's protocols. Chromatin isolation starts from 40 mg of tissue from the ascending part of the thoracic aorta. The tissue was washed using ice cold 1× PBS. After centrifugation at 1300 rpm/5 min/4°C, the supernatant was carefully removed and the pellet was resuspended thoroughly in 2 ml of 1× PBS containing of 1% formaldehyde for cross-linking the cells/for 8 min/37°C. Cross-linking was stopped by the addition of 125 mM of glycine. After centrifugation at 1300 rpm/5 min/4°C, the pellet was washed 5× with ice cold 1× PBS. The pellet was lysated with lysis buffer (LB consists of 155 mM NH4Cl, 10 mM KHCO3, and 0.1 mM EDTA pH 7.4) on ice for 10 minutes with gentle mixing. After centrifugation at 1500 rpm/5 min/4°C, the cell lysates were sonicated with a Bioruptor (Diagenode, Denville, USA) at high intensity for 5 min, with 30 s on/off intervals. For preclearing of chromatin, Protein G agarose beads (Merck Millipore, Prague, Czech Republic) were used for 1 hour/4°C. Chromatin fragments with lengths of 200 bp were visualized on agarose gel. Isolated cells were snap-frozen and stored at −80°C. A Nanodrop LC 3000 (Thermo Scientific, Bratislava, Slovak Republic) was used for measuring the concentration and purity of isolated chromatin, DNA, and RNA.

### 2.4. qRT-PCR

For detection of changes in the mRNA expression levels of specific genes *IL*-*6*, *hsCRP*, *TIMP*, *Emilin-1*, and *MMP9*, a Rotor-Gene Q-PCR thermocycler (Qiagene, Hilden, Germany) was used. RNA isolated from blood was transcribed into cDNA by using specific reverse primers individually for each gene and an M-MLV reverse transcriptase kit (Sigma-Aldrich). We also isolated chromatin from tissue and detected transcription activity of RNA Pol II, Emilin-1, and MMP9 using qRT-PCR methods after chromatin immunoprecipitation. In all, triplicated analyses were performed for each gene. Selected experimental genes (*IL*-*6*, *hsCRP*, *TIMP*, *Emilin-1*, and *MMP9)* and control housekeeping genes (*HPRT*, *ETNK*, *and GAPDH)* were amplificated by using 34 cycles (95°C/5 min, 95°C/15 s, 58–62°C/20 s, and 72°C/25 s) using appropriate specific primer sequences (Table 2). More detailed primer sequences are in Supplementary Table 5 available online at https://doi.org/10.1155/2017/9185934. Numerical quantification of changes in the expression of mRNA levels was evaluated by the comparative quantification and Ct value Q Rotor gene Software. The determination starts when, for each sample, difference between ΔCt of studied gene and control gene was calculated, then subtracted between ΔCt of sample with unknown concentration and ΔCt of the calibrator. The final result was a multiple of the calibrator concentration.

### 2.5. Copy Number Variation Analysis

Analysis of gene copies was performed after the isolation of DNA, using specific primers for all exon-specific gene domains of *IL*-*6*, *hsCRP*, *TIMP*, *Emilin-1*, and *MMP9* in comparison with HPRT and GAPDH. Amplification of specific genes was run for 33 cycles (95°C 5 min, 95°C for 15 seconds, 58°C–60°C for 20 seconds, and 72°C for 25 seconds) using the appropriate primer sequences with the Rotor-Gene Q-PCR (Qiagene, Hilden, Germany) thermocycler.

### 2.6. ChIP qRT-PCR

Precleared chromatin (125 ng) was incubated with selected antibodies (anti-RNAPII CTD YSPTSPS, anti-Emilin-1, and anti-MMP9, ab817, ab185953, ab 38898 (Abcam, Cambridge, UK) at 4°C overnight. The RNase treatment was done 30 minutes before incubation using a mix of RNase A/T (Roche Slovakia, Bratislava, Slovak Republic). Protein IgG agarose beads (Merck Millipore, Prague, Czech Republic) were used for bonding with immunocomplexes and cross-linking them. The next procedure used the downward line of cleaning buffer solutions in the order buffers I and II (500 mM NaCl, 50 mM HEPES (pH 7.5), 1% Triton-X-100, 0.1% sodium deoxycholate, and 1 mM EDTA (pH 7.5)), buffer III (10 mM Tris-Cl, 250 mM LiCl, 0.5% NP-40 (pH 8.0), 0.5% sodium deoxycholate, and 1 mM EDTA (pH 7.5)), and buffer IV (1 mM EDTA, 10 mM Tris-HCl). Immunoprecipitated DNA was eluted from the beads in TE Tris-EDTA buffer with 1% SDS. For reverse cross-linking of the samples, a solution was used containing 5 mol/l NaCl, 5 g/ml of enzyme RNaseA (Roche Slovakia, Bratislava, Slovak Republic), 1 M Tris-HCL (Sigma-Aldrich, Bratislava, Slovak Republic), and 20 g of proteinase K (Roche Slovakia, Bratislava, Slovak Republic), which was incubated at 65°C overnight and purified using a Qiagen PCR purification columns kit (28104, Qiagene, Hilden, Germany). DNA was eluted twice with 30 *μ*l of RNAase/DNAase free water (Qiagene, Hilden, Germany). An aliquot of 2 *μ*l of each sample was used for qRT-PCR using SensiMix (Bioline, Luckenwalde, Germany). Amplification was performed on a Qiagene Rotor-Gene Q-PCR thermocycler using the protocol: 30 cycles (95°C for 5 min, 95°C for 15 s, 60°C for 20 s, and 72°C for 25 s). All primer pairs (Sigma-Aldrich, Bratislava, Slovak Republic) used for ChIP analysis were designed using the Internet databases (http://www.genome.ucsc.edu/ and http://www.bioinformatics.org/sms/rev_comp.html). The primer pairs are listed in Table 2.

### 2.7. Protein Analysis by Randox Biochip

For the detection of protein levels in the serum of both the experimental and control groups, a Cytokine Array IV assay kit was used in combination with a biochip analyzer (Evidence Investigator, Randox Laboratories Ltd., London, UK). Detection of proteins IL-6, IL-6R, hsCRP, MMP9, TNFR1, and TNFR2 started with the incubation of a sample with 200 *μ*l of assay buffer for 1 hour/37°C/370 rpm of 100 *μ*l. After incubation, the procedure continued by decantation of the liquid and the washing of each well 2 times. The second incubation using the same conditions continued after adding conjugation buffer. After the second incubation, another decantation of liquid and the washing of each well 4 times were done. A mixture of luminol-EV-70l together with hydrogen peroxide was added to each well and incubated for 2 minutes. Visualization and calculation of the proteins levels (ng/ml) of each biomarker were performed using the Evidence Investigation biochip software version 4.

### 2.8. Statistical Analysis

All values are expressed as means ± SD for normally distributed data. Differences in proportions of categorical variables were analysed using Pearson chi-squared test, and continuous variables with normally distributed values were analysed using the Student *t*-test, whereas nonnormally distributed continuous data were analysed with Mann–Whitney *U*-test for two independent samples and Kruskal-Wallis test for more than two independent samples. Possible associations between aneurysm diameter and selected markers were evaluated by Pearson correlation test. Relationship between progression of aneurysm and selected markers was evaluated by linear regression. The level of statistical significance was set at *p* < 0.05. All analyses were performed using IBM SPSS 22.00 statistical software package.

## 3. Results

During the analysis of demographic data impact on the formation and progression of the aortic aneurysm, there were no any significant correlations found. We found that there is no difference in mean age between groups divided by aortic diameter. Differences are not statistically significant (rs = 0.293, *p* = 0.084). Due to the low number of women, there is no statistically significant difference in the male/female ratio in the individual aortic diameter categories (rs = 0.240, *p* = 0.070). Other demographic data (obesity, smoking, hypertension, and diabetes) also showed no significant correlation with aortic diameter with *p* > 0.05.

### 3.1. Changes in the Levels of Cytokines and Their Receptors

**Figure 1:**
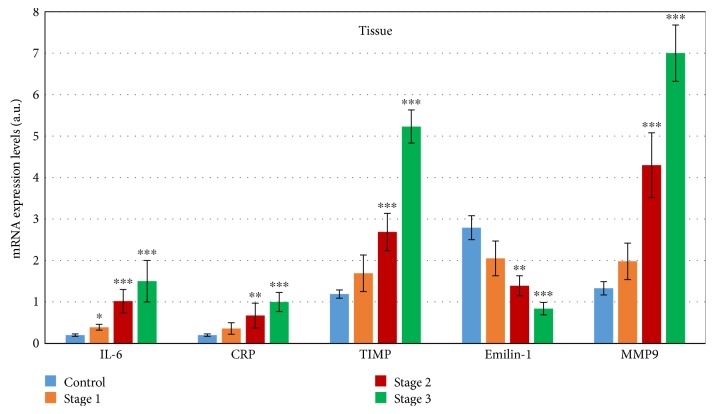
Expression of marker genes on mRNA levels in tissue of the patients with different stages of TAA. The mRNA levels of all detected genes were compared to controls (C, *n* = 10). All data are presented as average ± SD: 1—mean aortic diameter 43 ± 2.3 mm (*n* = 10), 2—mean aortic diameter 51 ± 2.8 mm (*n* = 34), and 3—mean aortic diameter 59.5 ± 3.7 (*n* = 14). ^∗^*p* < 0.05, ^∗∗^*p* < 0.01, and ^∗∗∗^*p* < 0.001 mean statistical significance. Maximal levels reached values about 400% higher than controls (*p* < 0.001).

The real-time PCR for IL-6 mRNA (Figure 1) in aortic tissue showed increased expression rising from stage 1 to the maximum in stage 3 (650% higher, *p* < 0.001) versus aortic controls.

**Figure 2:**
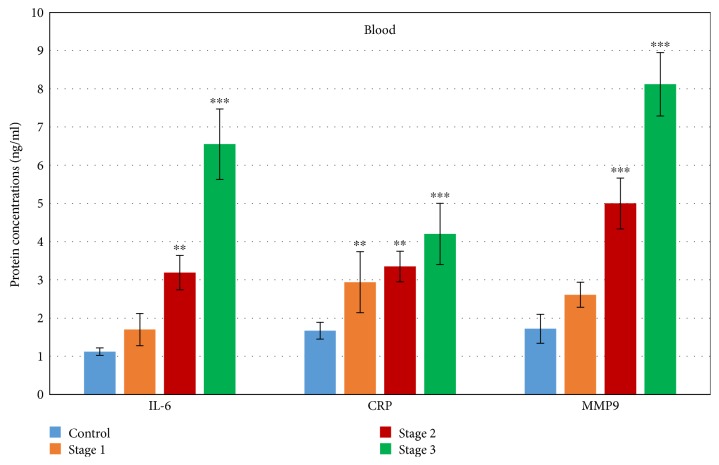
Expression of marker genes on protein levels in the blood of patients with different stages of TAA. The protein levels of all detected genes were compared to controls (C, *n* = 10). All data are presented as average ± SD: 1—mean aortic diameter 43 ± 2.3 mm (*n* = 10), 2—mean aortic diameter 51 ± 2.8 mm (*n* = 34), 3—mean aortic diameter 59.5 ± 3.7 (*n* = 14)., ^∗∗^*p* < 0.01 and ^∗∗∗^*p* < 0.001 mean statistical significance.

Immunochemical evaluation (Figure 2) of the blood serum showed a similar increase in the levels of final protein IL-6.

According to the Spearman correlation coefficients (nonparametric correlation), a strong correlation exists between the rising expression of IL-6 mRNA and increased aorta diameter (Table 3). Linear regression showed using categorical variable aorta diameter as a dependent variable that if the aorta diameter increases about one then an expression of IL-6 mRNA in tissue will be elevated about 0.710 (*p* < 0.001). Serum protein IL-6 did not correlate with the aorta diameter. The detection of mRNA levels of CRP in the aortic tissue (Figure 1) showed almost exponential growth from stage 1 to stage 3 against the control tissues.

Protein levels of CRP in blood serum (Figure 2) did not increase so dramatically. We found a maximal increase in stage 3 (about 151% times higher, *p* < 0.001) in comparison with the control serum.

We also demonstrated that the mRNA of CRP is produced in aneurysmal tissue, and its rising concentrations are associated with aneurysmal size, proved by Spearman correlation (Table 3).

**Figure 3:**
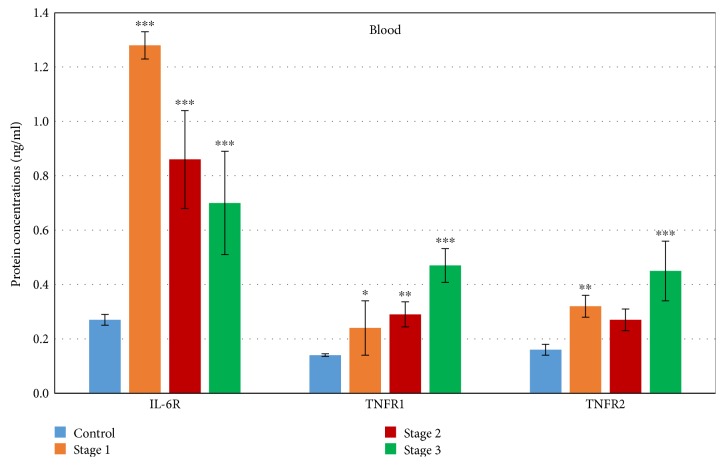
Expression of cytokine receptors on protein levels in blood of patients with different stages of TAA. The protein levels of all detected genes were compared to controls (C, *n* = 10). All data are presented as average ± SD: 1—mean aortic diameter 43 ± 2.3 mm (*n* = 10), 2—mean aortic diameter 51 ± 2.8 mm (*n* = 34), 3—mean aortic diameter 59.5 ± 3.7 (*n* = 14). ^∗^*p* < 0.05, ^∗∗^*p* < 0.01, and ^∗∗∗^*p* < 0.001 mean statistical significance.

Serum protein CRP was not correlated with the aorta diameter, because its levels could be affected by multiple factors related to nonspecific inflammation or infection and not only by aortic tissue damage. The most significant differences in protein levels of the cytokine receptors in blood were detected using antibodies for IL-6 receptor, where we found decreasing levels of protein from stage 1 with 374% higher levels than the control (*p* < 0.001) to almost 159% higher against the control in stage 3 (*p* < 0.001) **(**Figure 3**)**.

In contrast to these data, the protein levels of both TNFR receptors (TNFR1 and 2) were significantly elevated to the maximum in stage 3 **(**Figure 3**)**, with values about 230% and 181% higher than controls (both with *p* < 0.001).

During the statistical evaluation of Spearman correlation data, we found medium negative correlation between the aortic aneurysm diameter and protein levels of IL-6R (rs = −0.37, *p* < 0.024). The protein levels of TNFR1 positively correlated nonsignificantly with the progress of TAA (rs = 0.272, *p* < 0.058). All data are shown in Table 3.

### 3.2. Changes in the Expression of Components of ECM

The expression of Emilin-1 in aortic tissue on mRNA levels revealed a nonsignificant decrease from 28% less than control in stage 1 to a 70% smaller value against controls (*p* < 0.001) in stage 3. Linear regression showed using categorical variable aorta diameter as a dependent variable that if the aorta diameter increases about one then an expression of Emilin-1 mRNA in tissue will be decreased about 0.533 (*p* < 0.001). The pathological changes in ECM were confirmed by the detection of mRNA for MMP9 and TIMP (Figure 1). We found that the increase in mRNA levels of MMP9 (with the maximum in stage 3 about 490% higher than controls, *p* < 0.001) also affected the expression of TIMP; the mRNA levels of which were also elevated. Linear regression showed using categorical variable aorta diameter as a dependent variable that if the aorta diameter increases about one then an expression of MMP9 mRNA in tissue will be elevated about 0.193 (*p* < 0.001). Maximal levels of TIMP mRNA were detected in stage 3, with the value about 340% higher than in controls (*p* < 0.001). Linear regression showed using categorical variable aorta diameter as a dependent variable that if the aorta diameter increases about one then an expression of TIMP mRNA in tissue will be elevated about 0.280 (*p* < 0.001). The MMP9 protein levels in the blood were measured in the time-course of the growing diameter of the thoracic aorta aneurysm. We found that protein levels of MMP9 were nonsignificantly elevated from the initial stage of TAA (52% higher than controls) to highly significant levels about 372% higher in stage 3 (*p* < 0.001).

Spearman correlation of aneurysm diameter revealed a weak positive correlation in the mRNA of MMP9 (rs = 0.386, *p* < 0.048), which was also confirmed by the weak positive correlation of TAA progress with the blood level of protein MMP9 (rs = 0.320, *p* < 0.045). In agreement with this data, we found a medium positive correlation in mRNA levels of MMP9 and inhibitor TIMP (rs = 0.470, *p* < 0.025). The other marker of ECM degradation progress, Emilin-1, showed medium negative correlation with an aneurysm diameter on the mRNA level (rs = −0.496, *p* < 0,015). All data are shown in Table 4.

### 3.3. Results of Spearman Correlation Analysis

**Figure 4:**
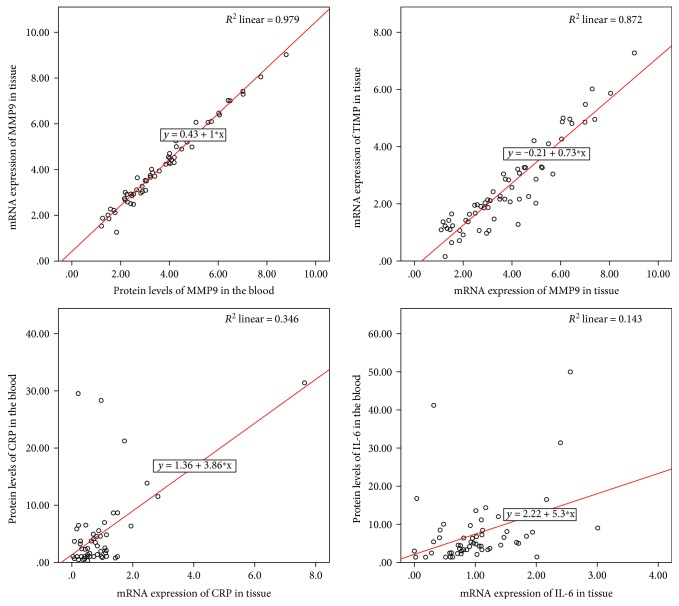
Graphical expression of the Spearman correlation of selected cytokines and markers of ECM damage.

The mRNA levels of both IL-6 and CRP had a similar rising ratio in the aortic tissue in all stages, which was also confirmed in the levels of both proteins in blood serum, where the IL-6 concentrations were about 160% higher than the levels of CRP (*p* < 0.001). During the statistical analysis of obtained results, we found a highly significant positive correlation between the expressions of mRNA of IL-6 in aortic tissue and protein levels of IL-6 in the blood (rs = 0.449, *p* < 0.01). A similar statistically significant positive correlation (Figure 4) was found between the mRNA expression of CRP in aortic tissue and protein levels of CRP in the blood (rs = 0.394, *p* < 0.01).

All of the results showed a strong positive correlation between mRNA levels of MMP9 and its protein MMP9 in blood (rs = 0.989, *p* < 0.001), which suggests that the progressive pathological changes of the TAA tissue cause the release of MMP9 into the blood of patients. This fact is also supported by the strong positive correlation between mRNA levels of TIMP and MMP9 in the tissue (rs = 0.934, *p* < 0.01, Figure 4). Linear regression revealed that if the level of MMP9 mRNA in tissue will be elevated about one then the mRNA of TIMP will be increased about 1.188 (*p* < 0.001). Because of the inhibitory activity of TIMP on MMP9, the imbalance between these genes is considered to be important in the degenerative process. Linear regression showed using categorical variable MMP9 mRNA as a dependent variable and protein MMP9 as an independent variable that if concentration of mRNA in tissue increases about one then the level of protein MMP9 in blood will be elevated about 0.013 (*p* < 0.051). Similarly to that also linear regression showed using IL-6 mRNA as a dependent variable and protein IL-6 as an independent variable that if concentration of mRNA in tissue increases about one then the level of protein IL-6 in blood will be elevated about 0.021 (*p* < 0.022).

## 4. Discussion

The occurrence and expansion of an aneurysm probably depend on local hemodynamic factors and intrinsic factors of the affected arterial segment. The medial layer of the aorta wall is responsible for its tensile elasticity and strength. During the formation of thoracic aorta aneurysm, elastin content in the ECM of the ascending aorta is progressively degrading. The activity and gene expression of specific enzymes (TGF, MMP) are increased, and this leads to the degradation of the structural proteins (elastin, fibullin, and collagen). Elastic fibre fragmentation and loss together with degeneration of the media result in a weakening of the aortic wall, loss of elasticity, and consequent dilation [19].

One of the main members of ECM is Emilin-1 [20]. Emilin-1 has multiple roles, like inhibiting elastin deposition by smooth muscle cells (SMC) [21] as well as regulation of the bioavailability of TGF-*β* by inhibiting proteolysis of the proTGF-*β* precursor to LAP/TGF-*β*, a complex from which the growth factor can be subsequently released for receptor binding [22]. The absence of Emilin-1 causes a remarkable increase of active TGF-*β*, which consequently through the SMAD cascade upregulates genes involved in ECM destruction (MMP9) or decreases SMC proliferation (p27) [23]. The importance of Emilin-1 expression was also confirmed by the experiment of Pivetta et al. [10], which showed that mice deficient in *Emilin-1* had increased TGF-*β* activity; however, these mice had a low incidence of aneurysms and no dissection. Another study, by Lee et al. [24], however, showed that embryonic mutation of the type II TGF-*β* receptor gene (*Tgfbr2*) impaired elastogenesis and resulted in aneurysm formation and inflammation. We found that the increasing diameter of aortic aneurysm significantly correlates negatively with decreasing levels of Emilin-1 mRNA in the affected tissue, which confirmed the direct involvement of Emilin-1 in the regulation of degradation processes of ECM in the aortic wall.

Another impairment in the maintaining of physiological conditions of ECM in aortic wall is upregulation of the expression of MMPs, which are defined as proteases produced by leukocytes and smooth muscle cells (SMCs) within the aortic wall and acting on a variety of extracellular protein substrates [25]. Specific MMP9 degrades type IV collagen, elastin, and various basement membrane proteins of SMCs. Its expression increases within 3 hours of the onset of dissection [11]. Ruddy et al. [26] observed that the activity of protein MMP9 in the aortic tissue of patients with Marfan syndrome, detected by zymography, was increased in comparison to controls. They also found that the concentration of protein MMP9 in the blood was significantly elevated as compared to controls. Gene expression analyses in animal models made by Trollope et al. [27] demonstrated the upregulation of the mRNA encoding MMP9 corresponding to increased extracellular matrix degradation. The results of Swedenborg et al. [28] have determined that the main producers of MMP9 in normal human aorta are mast cells. They found a higher amount of MMP9 in comparison to their expression in atherosclerosis, which demonstrates their involvement in extracellular matrix degradation, smooth muscle cell apoptosis, renin-angiotensin system activity, and neovascularization. In agreement with those results, we detected significantly elevated levels of both mRNA in aortic tissue (about 490% higher than controls in stage 3) and protein levels (with a maximum in the same stage of about 340% higher than controls). According to the statistically strong correlation between mRNA, protein levels of MMP9, and aortic diameter, we suggest that MMP9 expression was shown to be directly dependent on aneurysm diameter. For more precise confirmation, we measured the expression of mRNA for TIMP.

TIMP1 inhibits the activities of all MMPs and plays a role in regulating ECM in different physiological processes [26]. Structure function studies have separated the MMP inhibitory activity of TIMP1 from its growth promoting effect [29]. These TIMPs can express MMP-dependent and MMP-independent actions in the regulation of cell death, cell proliferation, and angiogenesis, involving specific signal transduction pathways. Several studies exist describing the ratio of MMP9 to TIMP1 expression in TAA. The study of Mi et al. [30] revealed that the ratio of MMP9 to TIMP1 in TAA tissue was 3.7-fold higher in TAA compared to controls. Another study of Ikonomidis et al. [31] confirmed the ratio of MMP9/TIMP1 was over 3.5-fold greater than controls. We found elevated mRNA levels of TIMP1 in all stages of TAA (with a maximum in stage 3, with levels about 340% higher than controls). We also correlated the mRNA ratio of MMP9 and TIMP and confirmed 1.7-fold higher values in aortic tissue against controls. Therefore, we suggest that an imbalance between MMP and TIMP expression is responsible for the shift toward a proteolytic state of ECM.

SMC apoptosis and ECM destruction in the aortic wall are accompanied by an increased degree of inflammation [11]. Several cytokines and chemokines that promote the recruitment of inflammatory cells to the aortic wall, such as tumour necrosis factor *α*, interferon (IFN)-*γ*, and interleukins IL-2 and IL-6 are upregulated [12]. These findings indicate that damage to the ECM, resulting in elastic fibre fragmentation, can trigger an inflammatory process by recruiting, activating, and inducing the differentiation of immune cells.

A classical plasma protein marker of acute phase of inflammation, infection, and tissue damage is CRP [32]. CRP is mainly expressed by hepatocytes, and its synthesis is regulated at the posttranscriptional level by cytokines, mainly by IL-6 with a synergic effect of IL-1 [13]. CRP can also be produced locally in atherosclerotic lesions [33]. A study by De Haro et al. [34] showed that patients with symptomatic and ruptured aneurysms had elevated serum CRP compared with patients with asymptomatic AAA. CRP directly influences several phases of atherosclerosis via complement activation, apoptosis, vascular cell activation, monocyte recruitment, lipid accumulation, and thrombosis [35]. CRP was normal and increased significantly since day 2 in the impaired oxygenation group [36]. Increased admission CRP correlated with high mortality irrespective of management policy [37]. However, Sakakura et al. [38] proposed that it may take 3–6 days to reach peak CRP; thus, initial CRP levels might not reflect the whole severity of aortic dissection. Our findings showed a rising concentration of both CRP mRNA in the tissue and also protein CRP in the blood of patients with a growing size of aortic diameter. Therefore, we supported the increasing inflammation in the TAA tissue and its spreading into the bloodstream, which was confirmed by elevated levels of IL-6 protein and mRNA in the blood and tissue.

IL-6 is involved in acute and chronic inflammation associated with aneurysm formation [39]. Both thoracic and abdominal aortic aneurysms are positively correlated to high circulating levels of IL-6 [13]. We found that soluble IL-6 in the samples with the highest aortic diameter had levels elevated by about 84% in comparison to controls. Our results are also confirmed by the study of Dawson et al. [40]. They demonstrated that TAA is a source of IL-6 in circulation, which was also demonstrated in the study of Golledge et al. [41], which showed that IL-6 values in AAA patients increased in a stepwise fashion among groups of aortic size and peaked in patients with aortic dilatation. This result confirmed that the aneurysm tissue is the source of the soluble IL-6, which is probably one of the key factors required for promoting Th17 cell differentiation; thus, one of the possible mechanisms of IL-6 action could be the regulation of Th17 cells in progression of TAA.

Another marker of increased inflammation enhanced by aortic dilatation is IL-6 receptor, which forms a dimer with the ubiquitously expressed signal transducer glycoprotein-130 (gp-130). Attachment of IL-6 to its receptor leads to the activation of the intracellular receptor-associated kinases and downstream effects via the transcription factor STAT3. The membrane-bound IL-6R (mIL-6R) is expressed in hepatocytes and cells of the innate immune system. In transsignalling, IL-6 binds to the circulating soluble IL-6R (sIL-6R), and this complex is capable of binding to gp130 in a wide range of cell types [42]. It has previously been shown that the expression of IL-6 and downstream mediators of IL-6 signalling, such as STAT3, are greater in AAA than in nonaneurysmal aortic tissue [32]. Till now, no data exists comparing the soluble protein of IL-6R concentrations and the diameter of TAA. We found a medium negative correlation of IL-6R with the diameter of aorta aneurysm.

We also studied the effect of the inflammatory cytokine TNF-*α* according to the expression of its receptors in the blood. TNF-*α* initiates its biological actions by binding to a 55-KDa receptor (TNFR1) or a 75-KDa receptor (TNFR2) [43]. TNFR1 is constitutively expressed in most tissues, binding primarily to the soluble form of TNF-*α*, and is the key mediator of TNF-*α* signalling in many cell types. TNFR2 is typically expressed in endothelial- and immune-related cells and is activated by membrane-bound TNF-*α*. The major difference between the two receptors is the death domain (DD) of TNFR1 that is absent in TNFR2. Meng et al. [44] suggest that during early stages of aneurysm formation, TNFR2 signalling is activated by membrane-bound TNF-*α* and when sufficient TNF-*α* is secreted, it activates TNFR1 signalling, resulting in inflammation and apoptosis [45]. We found that both TNFR receptors (TNFR1 and TNFR2) had protein levels significantly elevated to the maximum in the group of patients with the higher aneurysm diameter. This confirms the nonsignificantly positive correlation of TNFR receptors with the progress of TAA.

## 5. Conclusion

The asymptomatic progress of TAA predetermines the detection of early pathological changes in aortic tissue as one of the most important goals of current cardiovascular treatment. Recently used detection techniques (ultrasonography and MRI) have very high specificity and efficiency. However, the possibility of repeating measurements during the control examination is limited regardless of the time occupancy of individual scanning equipment. Therefore, the aims of this paper were focused on the detection of basic inflammatory markers as well as markers of ECM degradation in both the aortic wall and blood. From the obtained results, a positive correlation of parameters like IL-6, CRP, TNFR1, and TNFR2 together with MMP9 and TIMP between the growing diameter of aneurysm and mRNA from tissue or protein levels from blood is obvious, although we also found significant negative correlation of Emilin-1 mRNA in the tissue and protein soluble receptor of IL-6 in the blood, which suggests that the release of inflammatory mediators dramatically increases the degradation of ECM in the aortic wall. Changes of gene expression of selected genes can be used for more purposes, like the detection of progressive pathological changes of aortic wall, for the experimental study of the inflammatory receptor inhibitors or effect of the gene polymorphism on the receptor functions during trials targeted on slowing down the progress of aortic wall aneurysm or for a decision about the consequential surgical options of wall recovery.

## Supplementary Material

Supplementary Table 5. List of used primers sequences (more detailed version of sequences).

## Figures and Tables

**Table 1:** Demographic and clinical characteristics of subjects.

	Control	Stage 1	Stage 2	Stage 3
Number of patients	35	12	34	14
Gender (female %)	20/35 (57%)	2/12 (17%)	5/34 (14.7%)	2/14 (14.3%)
Age (years)	45 ± 9.6	49.9 ± 16.6	55.1 ± 14.7	54.5 ± 14.1
Aorta diameter (mm)	—	43 ± 2.3	51 ± 2.8	59.5 ± 3.7
AR/AS	—	6/6 (50/50%)	19/15 (56/44%)	8/6 (57/4%)
BAV/TAV	—	8/4 (67%)	21/13 (62%)	8/6 (57%)
Hypertension	5/35 (14%)	8/12 (67%)	18/34 (52.9%)	12/14 (85.7%)
Diabetes mellitus	0	1/12 (8%)	3/34 (8.8%)	2/14 (14.3%)
Current smokers	9/35 (26%)	7/12 (58.3%)	22/34 (64.7%)	11/14 (78.6%)
BMI	26.4 ± 4.2	29.4 ± 6.4	29.6 ± 4.9	26.99 ± 4.9
Weight (kg)	76.9 ± 15.2	89.2 ± 16.9	89.3 ± 13.7	87.4 ± 16.3

**Table 2:** Localization of the chromosome of specific genes (http://www.genome.ucsc.edu).

Name of gene	Chromosomal localization	Size of gene in bp including UTR side	Analysis place of gene Ex-exon
IL-6	7p15.3	22, 766, 766–22, 770, 157	Prom, Ex1, Ex4
CRP	1q23.2	159, 682, 079–159, 684, 379	Ex1, Ex2
TIMP	Xp11.23	47, 441, 712–47, 446, 188	Ex1, Ex3
Emilin-1	2p23.3	27, 301, 435–27, 309, 265	Prom, Ex1, Ex3, Ex4, Ex6
MMP9	20q13.12	44, 635, 634–44, 647, 114	Prom, Ex1, Ex5, Ex9, Ex13
GAPDH	12p13.1	6, 643, 585–6, 647, 537	Ex2, Ex3, EX4
HPRT	Xq26.2-q26.3	133, 594, 175–133, 634, 698	Ex3, Ex6
ETNK	12p12.1	22, 778, 076–22, 843, 608	Ex1, Ex3, Ex7

**Table 3:** Spearman correlations and linear regression between the mRNA, protein levels, and aortic diameter for selected cytokines and their receptors.

	mRNA in aortic tissue		Protein levels in blood				
	CRP	IL-6	CRP	IL-6	IL-6R	TNFR1	TNFR2
Correlation coefficient	0.489^∗∗^	0.554^∗∗^	0.025	0.320	−0.37^∗^	0.272	0.268
*p* value	0.015	0.017	0.642	0.524	0.024	0.058	0.063
Unstandardized coefficient	0.105	0.710^∗∗∗^	0.027	−0.006	0.007	0.966	0.771
*p* value	0.197	0.000	0.051	0.503	0.004	0.177	0.120

Statistical significance: ^∗^*p* < 0.05, ^∗∗^*p* < 0.01, and ^∗∗∗^*p* < 0.001.

**Table 4:** Spearman correlations and linear regression between the mRNA, protein levels, and aortic diameter for a selected member of ECM.

	mRNA in aortic tissue			Protein levels in blood
	MMP9	Emilin-1	TIMP	MMP9
Correlation coefficient	0.386^∗^	−0.496^∗∗^	0.470^∗∗^	0.320^∗^
*p* value	0.048	0.015	0.025	0.045
Unstandardized coefficient	0.193^∗∗∗^	−0.533^∗∗∗^	0.280^∗∗∗^	0.007
*p* value	0.000	0.000	0.000	0.004

^∗^ *p* < 0.05, ^∗∗^*p* < 0.01, and ^∗∗∗^*p* < 0.001 mean statistical significance.
