# Supplementary material for: Detection of Pathological Changes in the Aorta during Thoracic Aortic Aneurysm Progression on Molecular Level
Source: Dis Markers. 2017 Oct 12;2017:9185934. doi: 10.1155/2017/9185934 (PMC5660829; doi:10.1155/2017/9185934)
Supplement: Supplementary file 1 — Supplementary Table 5. List of used primers sequences (more detailed version of sequences). [file 9185934.f1.docx]

**Supplementary Table 5. List of used primers sequences (more detailed version of sequences)**

| **Name of gene** | **Loci** | **Forward sequence** | **Reverse sequence** | **Lenght (bp)** |
| --- | --- | --- | --- | --- |
| IL6 | Prom | CTTTGGTTTTTACAAATACAAAT | CTGAAGTCATGCACGAA | 135 |
| IL6 | Ex1 | TCTCAATATTAGAGTCTCAACCC | TAGAGCTTCTCTTTCGTTCC | 98 |
| IL6 | Ex4 | ACTTGCCTGGTGAAAATCAT | TCTGGCTTGTTCCTCACTACT | 96 |
| CRP | Ex1 | AGCTCCCTATCTGGAGGATAGTT | CAAAGTCCATAACTCAATCCTTG | 87 |
| CRP | Ex2 | AAAGCACCGTTAACGAAGC | AAAATACTGTACCCACGGGT | 90 |
| TIMP | Ex1 | AGTATATACAACTCCACCAGA | ATTACAGCTGATGTCAAAAAC | 147 |
| TIMP | Ex3 | AAAGGCCGAGGGGGAC | AGCCCATCTGGTACCTGT | 75 |
| Emilin-1 | Prom | TTGGACAAGTCACTCTCCC | TGGACAAGGGAGAATGAACT | 111 |
| Emilin-1 | Ex1 | TCATCAGGGAAAGAGGACA | TGTTCTCTGTGCCCCCT | 140 |
| Emilin-1 | Ex3 | TACAAGACAGTGACCGACAT | AAGACGCAGGCCCCA | 97 |
| Emilin-1 | Ex4 | ATGAAACCCTCAATGAGATC | TGATGGTTGTTGAGGTGA | 95 |
| Emilin-1 | Ex8 | TGGAGAATAAGCCGGTGG | ATGGTGAGCGGCTCCTC | 144 |
| MMP9 | Prom | AGACACCTCTGCCCTCAC | ATTGGTTCTCAGGTCTCCA | 133 |
| MMP9 | Ex1 | ATACCTGTACCGCTATGGTTACA | TGGATAGCGCCACGCT | 120 |
| MMP9 | Ex5 | AACTCGGTTTGGAAACGC | GGTCGTCGGTGTCGTAGTT | 143 |
| MMP9 | Ex9 | TGAACCTGAGCCACGGC | GCTCTGAGGGGTGGACAGT | 93 |
| MMP9 | Ex13 | TATTTCTGCCAGGACCGC | ACTGCAGGATGTCATAGGTCA | 97 |
| GAPDH | Ex1 | TTTCTATAAATTGAGCCCGCA | ACCTGGCGACGCAAAA | 87 |
| GAPDH | Ex8 | AACCTGCCAAATATGATGAC | AGGAAATGAGCTTGACAAAG | 168 |
| HPRT | Ex3 | AGATGTGATGAAGGAGATGG | AATAGCTCTTCAGTCTGATAAAATC | 164 |
| HPRT | Ex6 | TTGTTGGATTTGAAATTCCAG | ATTCAAATCCCTGAAGTATTCAT | 87 |
| ETNK | Ex1 | TCTAGCCCTTAAATGCACAC | TGAATCTTCACAGCAAACAG | 182 |
| ETNK | Ex3 | TTATACAAGGAGAAGCACTGG | ACAAAAACAAAAACCCTAGC | 196 |
| ETNK | Ex7 | ATTTGCTCATGCAAAAACTC | AAATGTTTCAAACCCTCAAG | 201 |
